# Pharmacokinetic variability of everolimus and impact of concomitant antiseizure medications in patients with tuberous sclerosis complex: A retrospective study of therapeutic drug monitoring data in Denmark and Norway

**DOI:** 10.1097/MD.0000000000039244

**Published:** 2024-08-09

**Authors:** Kjersti Kirkeby, Ine Cockerell, Jakob Christensen, Christina Engel Hoei-Hansen, Lotte Holst, Mikkel G. Fredriksen, Caroline Lund, Cecilie Johannessen Landmark

**Affiliations:** a Department of Pharmacy, Faculty of Health Sciences, Institute of Life Sciences and Health, Oslo Metropolitan University, Oslo, Norway; b Department of Rare Disorders and Disabilities, National Centre for Rare Epilepsy-Related Disorders, Oslo University Hospital, Oslo, Norway; c Department of Neurology, Aarhus University Hospital, Affiliated Member of the European Reference Network EpiCARE, Aarhus, Denmark; d Department of Pediatrics, University Hospital Rigshospitalet, Copenhagen, Denmark; e Department of Clinical Medicine, University of Copenhagen, Copenhagen, Denmark; f Department of Neurohabilitation, Oslo University Hospital, Oslo, Norway; g The National Centre for Epilepsy, Member of the ERN EpiCare, Oslo University Hospital, Oslo, Norway; h Department of Pharmacology, Section for Clinical Pharmacology, Oslo University Hospital, Oslo, Norway.

**Keywords:** enzyme inducers, interactions, long-term therapeutic drug monitoring

## Abstract

The mTOR-inhibitor everolimus is a precision drug with antiepileptogenic properties approved for treatment of epilepsy in persons with tuberous sclerosis complex (TSC) in combination with other antiseizure medications (ASMs). However, the pharmacokinetic variability of everolimus is scarcely described, and the available information on pharmacokinetic interactions is scarce. The purpose of this study was to investigate pharmacokinetic variability of everolimus in patients with TSC, and the impact of age, sex and comedication. In this retrospective observational study we used anonymized data from medical records of patients with TSC using everolimus in Norway and Denmark, 2012 to 2020. Long-term therapeutic drug monitoring (TDM) identified inter-patient and intra-patient variability. The study included 59 patients, (36 females (61%)), median age 22 (range 3–59 years). Polytherapy was used in 50 patients (85%). The most frequently used ASMs were lamotrigine (n = 21), valproate (n = 17), and levetiracetam (n = 13). Blood concentrations of everolimus were measured in all patients. Pharmacokinetic variability of everolimus between patients was extensive, as demonstrated by a 24-fold variability from minimum–maximum concentration/dose (C/D)-ratios. The coefficient of variation (CV) for intra-patient (n = 59) and inter-patient variability (n = 47, ≥3 measurements) was 40% and 43%, respectively. The C/D-ratio of everolimus was 50% lower in 13 patients (22%) using enzyme-inducing ASMs compared to the 30 patients who did not (0.7 vs 1.4 ng/mL mg, *P* < .05). Age and sex were not significantly associated with changes in C/D-ratios of everolimus. Long-term TDM identified extensive variability in concentrations over time for everolimus both within and between patients, where comedication with enzyme-inducing ASMs was an important contributing factor. The findings suggest a need for TDM in patients with TSC treated with everolimus.

**Highlights**

- Everolimus, mTOR-inhibitor, is a precision antiepileptogenic drug in tuberous sclerosis complex (TSC) and epilepsy
- Long-term TDM demonstrated extensive variability in blood concentrations over time for everolimus
- Comedication with enzyme-inducing ASMs contributed to intra-patient and inter-patient variability

## 1. Introduction

Tuberous sclerosis complex (TSC) is a rare, autosomal dominant genetic disorder causing benign tumors in different organs, such as the brain, kidneys, lungs, and heart, and about 80% develop focal epilepsy.^[1,2]^ Early onset epilepsy is associated with an increased risk of neurodevelopmental disorders, such as autism spectrum disorders and intellectual disability.^[1]^ Many TSC patients have refractory epilepsy, and polytherapy with antiseizure medications (ASMs) is common, increasing the risk of drug interactions. Everolimus is a selective inhibitor of the mTOR-signaling pathway and consequently, of growth and proliferation of cells involved in TSC, in addition to reduction of glutamatergic excitation.^[3]^ Thus, everolimus is considered a precision drug with antiepileptogenic properties.^[4–6]^ The efficacy of everolimus for focal seizures in patients with epilepsy secondary to TSC was demonstrated in a randomized clinical trial (EXIST-3).^[4]^ Everolimus is approved as adjunctive treatment for patients from 2 years with TSC and refractory focal seizures.^[7]^

The use of therapeutic drug monitoring (TDM) may help to individualize drug treatment by adjusting dosages to account for pharmacokinetic variability related to age, sex and use of comedication.^[8–12]^ Long-term TDM in patients with multiple drug measurements has recently been used as a tool to investigate intra-patient and inter-patient pharmacokinetic variability of ASMs used in specific epilepsy syndromes as juvenile myoclonic epilepsy and Dravet syndrome.^[13,14]^ There are few studies that focus on pharmacokinetic variability of everolimus in a clinical setting, but systematic surveillance is important due to a narrow therapeutic index and numerous adverse effects of everolimus.^[4]^

The purpose of the present study was therefore to investigate pharmacokinetic variability of everolimus in patients with TSC, and the impact of age, sex, and comedication with ASMs.

## 2. Methods

### 2.1. Patient material and laboratory data

In this observational study TSC patients using everolimus were included from 3 university hospital clinics and the National Centre for Rare Epilepsy-Related Disorders, Oslo University Hospital as described by Cockerell et al.^[15]^ Retrospective and anonymized data from medical records from May 2012 to January 2020 were collected. The following variables were included: sex, age at first concentration measurement, date of measurements, daily dose of everolimus, blood concentrations of everolimus, concomitant use, and doses of other ASMs. Body weight and use of other drugs than ASMs were not available and therefore not systematically noted for all patients.

The concentration measurements of everolimus were performed as routine analysis in whole blood with validated methods using LC-MS/MS methodologies at the Laboratories for Clinical Pharmacology at Oslo University Hospital, Aarhus University Hospital and Rigshospitalet University Hospital in Copenhagen. The reference range for everolimus for the indication SEGA and refractory epilepsy was 5 to 15 ng/mL, as provided in the product information.^[7]^

All samples of both everolimus and ASMs were drawn drug-fasting in the morning at steady state, as part of the standard procedure. The Norwegian Medical Ethics Committee reviewed and approved the study (Ethics Committee No. 2013/176-36). The Central Denmark Region Committees on Health Research Ethics declared the study exempted from notification, as a data processing contract was completed.

### 2.2. Calculations

All data of previous and present use of ASMs and blood concentration measurements were collected and included age, daily dose of everolimus, concentration of everolimus, and dose and serum concentration measurements of all other ASMs. Calculations included all TDM data available. Mean and median concentrations, doses, and concentration/dose (C/D)-ratios were calculated standard deviation (SD) or range to express variability. To study the impact of comedication on everolimus metabolism, concentration/dose ratio (C/D-ratios) from the most recent measurement of everolimus were calculated. Everolimus is metabolized via the CYP3A4/5 pathways, and based on theoretical potential for interaction via these pathways, we categorized the patients into the following groups:^[7,13,14,16,17]^ everolimus used in monotherapy or with concomitant use of ASMs that had no strong enzyme-inducing or enzyme-inhibiting properties on the CYP3A4/5 pathway (brivaracetam, clobazam, clonazepam, ethosuximide, gabapentin, lacosamide, lamotrigine, levetiracetam, nitrazepam, perampanel, pregabalin, rufinamide, topiramate, vigabatrin, and zonisamide), use of strong enzyme-inducing drugs with potential impact on the CYP3A4/5 pathway (carbamazepine, eslicarbazepine, oxcarbazepine, phenytoin, and phenobarbital); and use of valproate, which may have undefined properties on the CYP3A4/5 metabolic pathway. Patients with concomitant use of drugs with strong enzyme-inducing properties who also used valproate (i.e., groups B + C) were excluded from further analyses.

To study interindividual and intraindividual variability over time, C/D-ratios for individual patients with multiple measurements (3 or more) were included. A measure of overall pharmacokinetic variability was calculated by the difference between the minimum and maximum C/D-ratio as previously,^[13,14]^ and the coefficient of variation (CV) for C/D-ratios (standard deviation * 100/mean C/D-ratio) was calculated for each patient. The mean expressed as intra-patient variation to quantify the pharmacokinetic variability, based on previous studies.^[13,14,18,19]^ The CV expressing inter-patient variability was calculated based on the C/D-ratios for patients with 3 or more measurements.

### 2.3. Statistical analyses

For statistical analyses IBM SPSS Statistics version 25 was used. Student 2-sided *t* test with unequal variance was used for comparison of possible group differences for sex and age. A linear regression model (*r*^2^) was used to evaluate the relationship between drug dose and concentration for the 3 most used ASMs. Statistical significance between the 3 groups of comedications were evaluated by analysis of variance (ANOVA) for comedication and variance of CVs, by 1-way Anova, with Bonferroni corrections for multiple comparisons and Levene test of equality of error variances. *P* values of <.05 were considered statistically significant for all analyses.

## 3. Results

### 3.1. Patient and laboratory data

The patients in the present study were included from neurological and pediatric departments in Denmark and Norway with a similar distribution in age, sex, and use of ASMs between the countries (n = 59). There were 36 females (61%), median age 22 years (range 3–59 years), and mean age 23 years (SD 14.5) at the time of initiation of treatment with everolimus (Table 1). The mean number of everolimus measurements was 9 (SD 12.2), and the median number was 5.5 (range 1–76). There was no difference between those who had at least 10 measurements, as compared to those with less measurements (Table 1). Among the total of 59 patients, there were 9 patients who used everolimus in monotherapy, while 50 used 1 to 4 other concomitant ASMs. Those who used everolimus as monotherapy had the indication renal angiomyolipoma and not epilepsy. There were 30 patients who used everolimus in monotherapy or with neutral ASMs (group A), 13 who used everolimus with enzyme-inducing ASMs (group B), and 16 who used everolimus with valproate (group C).

**Table 1:** Patient characteristics.

Parameters	Mean (SD)/median (range)
Patients (n = 59) 33 from Norway/26 from Denmark	
Sex: 36 female/23 male	
Age at the time of data collection	23.4 (14.5)
	22 (3–59)
Total number of measurements	536
Number within the reference range (5–15 ng/mL)	285 (53%)
Number of measurements per patient	9 (12.2)
	5.5 (1–76)
Dose (mg)	5.51 (3.89)
	5.0 (0.5–34.0)
Blood concentration (ng/mL)	7.02 (4.66)
	5.9 (0.93–29.3)
C/D-ratio everolimus (ng/mL mg)	
All, independent of comedication (n = 59), 24-fold variability	1.37 (1.0)
	1.04 (0.2–4.7)
Use of monotherapy or neutral ASMs (n = 30)	1.39 (1.1)
	1.02 (0.2–4.7)
Use of enzyme-inducing ASMs (n = 13)	0.70* (0.48)
	0.58 (0.24–1.91)
Use of valproate (n = 16)	1.81 (0.96)
	2.0 (0.29–3.8)
C/D-ratios between sex	
Females (n = 36)	1.53 (0.94)
	1.21 (0.29–3.8)
Males (n = 23)	1.04 (1.04)**
	0.71 (0.2–4.7)
Interindividual and intraindividual variability	
Coefficient of variation (CV) total	39.97 (0.63)
CV: patients with ≥10 measurements (n = 17)	40.36 (0.64)
CV: patients with <10 measurements (n = 42)	39.75 (0.62)

CV = coefficient of variation.

The value at the latest measurement was used for each patient.

* Significant change from monotherapy/neutral antiseizure medications (ASMs) (*P* < .05).

** No significant difference.

### 3.2. Pharmacokinetic variability and impact of comedication

**Figure 1.**
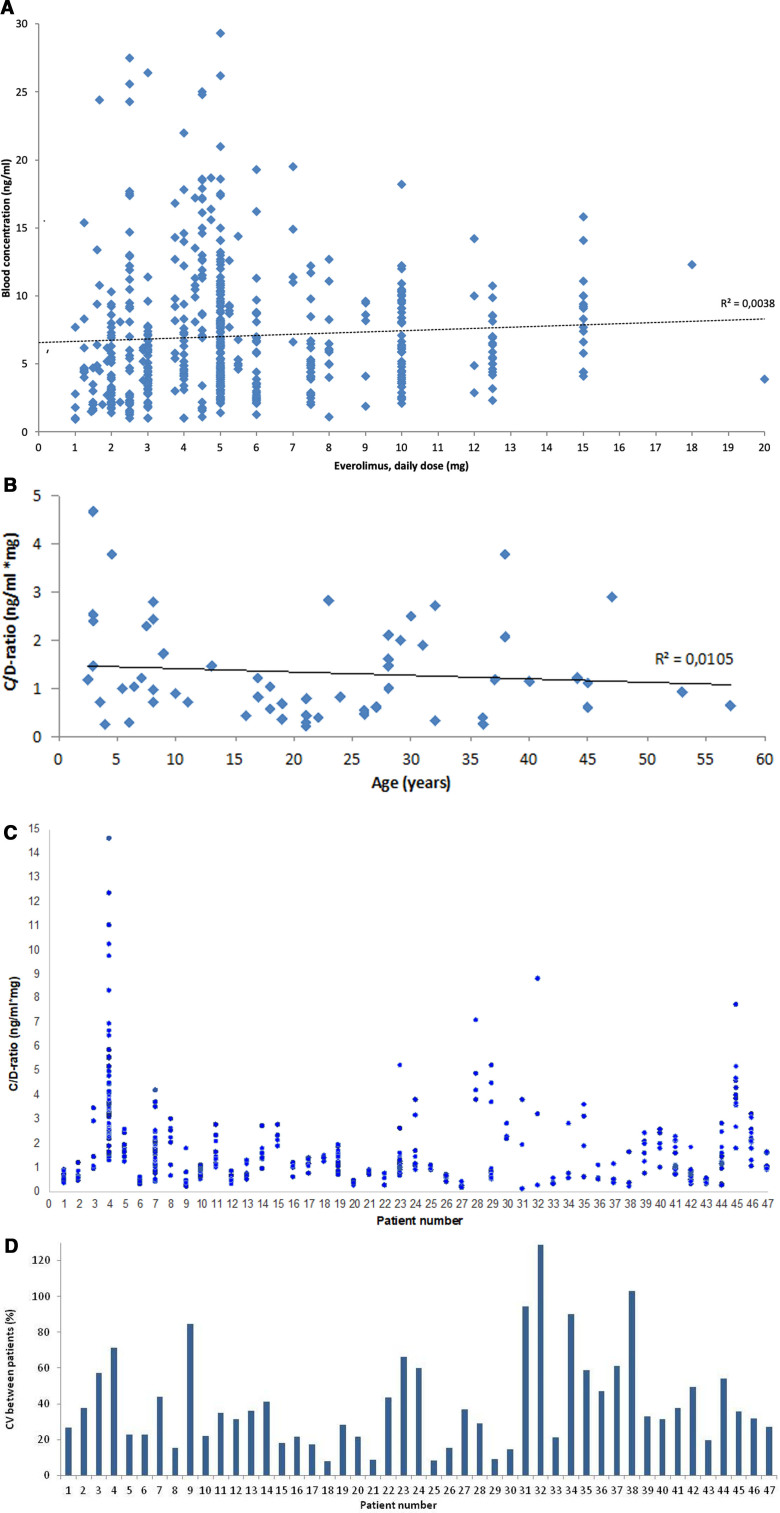
Pharmacokinetic variability of everolimus. (A) Dose and blood concentration relationships of everolimus, all measurements (n = 536) from all patients (n = 59) included. The solid horizontal lines illustrate the reference range for everolimus for the indication SEGA and refractory epilepsy: 5 to 15 ng/mL. The dotted line indicates the linear regression line. (B) C/D-ratio and age distribution, where the latest measurement for each patient was used (n = 59). The line indicates the linear regression line. (C, D) Intra-patient and inter-patient variability in C/D-ratio for patients with at least 3 measurements, as expressed by the coefficient of variability (CV) given in % for every patient. Each patient has a number at the *x* axis (n = 47). Patient #4 with an extensive number of measurements started and tapered an enzyme inducer, oxcarbazepine for 2 months in the beginning, leading to a decreased blood concentration, total follow-up period 7 years.

There was extensive variation of doses and concentrations of everolimus and across age groups (Fig. 1A and B). The C/D-ratios were similar between males and females (Table 1). There was no significantly linear correlation between age and C/D-ratio (n = 59) (*r*^2^ = 0.01) by using the most recent measurement. In the 47 patients with more than 3 everolimus measurements, pharmacokinetic variability was pronounced, expressed as a 24-fold variability in C/D-ratio between patients, and additionally intra-patient variability (40%) and inter-patient variability (43%) in C/D-ratios, shown with corresponding coefficients of variation (CVs) (Fig. 1C and D).

For instance, patient #4 used between 2 and 5 concomitant ASMs in addition to everolimus. The C/D-ratios varied between 1.31 ng/mL mg and 14 to 61 ng/mL mg (11-fold variation). The enzyme-inducing drug oxcarbazepine was used for 2 months in the initial phase of treatment, during which 3 concentration measurements were performed, everolimus decreased slightly, and oxcarbazepine was discontinued. Then, the patient’s concentration measurements were more stable the last 3 years of the time period, where valproate and 2 neutral ASMs in addition to everolimus were used.

Thirteen patients (22%) used CYP3A4/5 enzyme-inducing ASMs at the most recent concentration measurement, which was associated with a 50% lower C/D-ratio of everolimus, as compared to the non-inducer/inhibitor group (n = 30) (0.7 vs 1.4 ng/mL mg, *P* < .05). The dose was on average adjusted by only 15% in patients who tapered enzyme inducers (n = 8), mean dose 7.15 mg/day before versus 6.19 mg/day after tapering (group A). The C/D-ratio of everolimus in those who concomitantly used valproate (group C) (n = 16) was not significantly different from the non-inducer/inhibitor group (group A) (1.8 vs 1.4 ng/mL mg, respectively (*P* = .21)) (Table 1).

**Figure 2.**
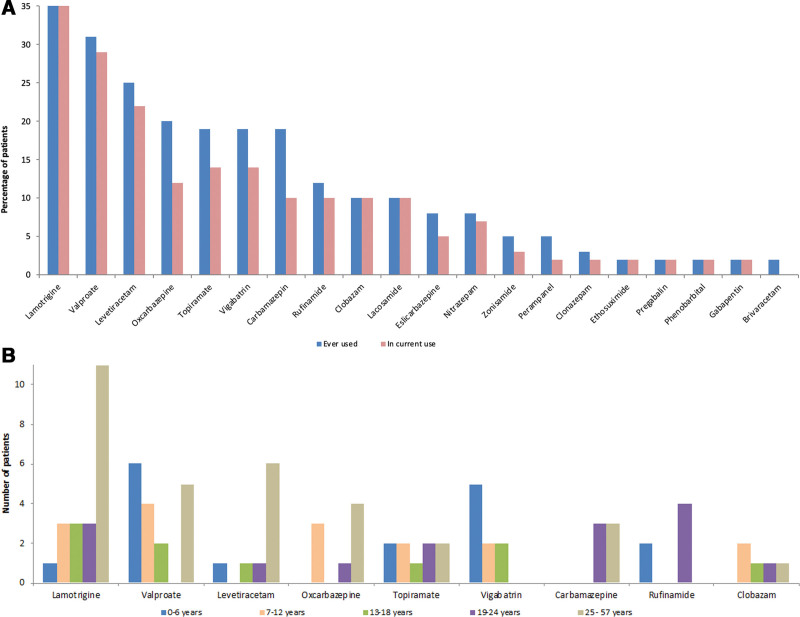
(A) Use of various antiseizure medications (ASMs) ever used or in current use with everolimus. Number of patients: lamotrigine (n = 22/21), valproate (n = 18/17), levetiracetam (n = 15/13), oxcarbazepine (n = 12/7), topiramate (n = 11/8), vigabatrin (n = 11/8), carbamazepine (n = 11/6), rufinamide (n = 7/6), clobazam (n = 6/6), lacosamide (n = 6/6), eslicarbazepine (n = 5/3), nitrazepam (n = 5/4), zonisamide (n = 3/2), perampanel (n = 3/1), clonazepam (n = 2/1), ethosuximide (n = 1/1), pregabalin (n = 1/1), phenobarbital (n = 1/1), gabapentin (n = 1/1), and brivaracetam (n = 1/0). (B) Use of ASMs as comedication in addition to everolimus in various age groups: 0 to 6 years (n = 11), 7 to 12 (n = 9), 13 to 18 (n = 6), 19 to 24 (n = 9), and 25 to 57 (n = 24). Age and use of ASMs at the latest measurement were used.

Figure 2A and B illustrates previous and current use of other ASMs, and the use of various drugs in different age groups. This adds important data regarding evaluation of comedication and the impact of possible interacting ASMs. Enzyme inducers were less used in the youngest children. It is also shown that for instance lamotrigine was mainly used among the adults, while vigabatrin was used more in the youngest age group. Lamotrigine, valproate, and levetiracetam were mostly used, in previous and current treatment. The use of the enzyme inducers oxcarbazepine and carbamazepine was lower at the most recent measurement than during the total time of treatment.

## 4. Discussion

The present study include a large number of TSC patients treated with everolimus and concomitant ASMs in Norway and Denmark. TDM was routinely applied in all patients, from frequent to more moderate intervals of follow-up. Long-term TDM revealed pronounced pharmacokinetic variability of everolimus over time and extensive use of polytherapy with ASMs which cause significant pharmacokinetic interactions with everolimus, such as the combination with enzyme inducers of the CYP3A4/5 pathways.

### 4.1. Use of ASMs

Most patients used everolimus with other ASMs; lamotrigine, valproate, and levetiractam were mostly used in combination with everolimus. These are also the most used ASMs in all patients with epilepsy in Norway, possibly also reflected in Denmark.^[20]^ Vigabatrin was used in the youngest patients as this is recommended as first- line therapy for infantile spasms, and in children below 1 year of age with focal seizures and is in line with recommendations.^[17,21]^ Interestingly, the use of enzyme-inducing ASMs such as carbamazepine and oxcarbazepine diminished over time during the period of data collection. It was less frequently used in the younger age groups. This interaction is well known, and use of concomitant enzyme-inducing ASMs will require higher doses of everolimus to achieve the same blood concentration as compared to use of concomitant non-inducing drugs. However, in this study the dose of everolimus was only adjusted moderately (15%) after switching from enzyme-inducing ASMs to non-inducing ASMs. This may reflect that those who used higher doses of everolimus did not achieve sufficiently high blood concentrations to experience efficacy due to the induction by enzyme-inducing ASMs. Thus, it seems rational to switch to non-inducing ASM comedications when using everolimus, taking other clinical considerations of efficacy and tolerability also into account.

### 4.2. Pharmacokinetic variability and impact of comedication

Extensive variability in C/D-ratios was demonstrated for everolimus. Comedication with enzyme inducers affected the C/D-ratios significantly, as previously shown, and this was expected based on the CYP3A4/5 pathway for metabolism of everolimus and being a substrate of P-glycoprotein.^[4,7,17,22]^ We revealed, however, that this pharmacokinetic interaction was not accounted for when individualizing the dose of everolimus. Concomitant use of ASMs that are enzyme inducers, but not sex and age were identified to contribute to variability in blood concentration of everolimus between patients. Valproate does not to affect the CYP3A4/5 pathway and was not associated with altered C/D-ratio of everolimus which is in line with a recent study of the impact of comedication with the ASM perampanel, which shares the same metabolic pathway as everolimus.^[23]^ In a recent study of 183 adult patients with TSC, 47% used polytherapy with ASMs, where pharmacokinetic interactions may be relevant.^[24]^ Previously, it has also been shown that age and sex did not have significant impact as single factors explaining pharmacokinetic variability of everolimus.^[22,25]^ In this study, it would have been relevant to use the C/D-ratio per kg body weight in children, as dose in children is correlated to body weight, but this parameter was not available for most patients. Franz et al^[17]^ demonstrated however, that the starting dose should be chosen according to age under or above 6 years to reach a TDM target range of a C_ss, min_ of 5 to 7 ng/mL (and up to 15 ng/mL if necessary). Stockinger et al^[26]^ recently concluded that everolimus is effective as add-on treatment in epilepsy in adults with TSC, with no age limit observed to gain an individual benefit. Recently, Cockerell et al^[15]^ also reported that there was no association between dose, blood concentration or C/D-ratios, and responders/nonresponders, based on a clinical evaluation and retention rates of the patients included in the present study. It was shown that one third of patients were defined as responders, with a mean concentration of 7.5 ng/mL and C/D-ratio 1.73 ng/mL/mg (SD = 1.06) (n = 12) in responders (>50% seizure reduction) versus nonresponders 5.9 ng/mL and 1.35 (SD = 1.01), respectively (n = 35).^[15]^ As recently documented, the target reference range of 5 to 15 ng/mL was aimed at, but may be difficult to achieve due to tolerability or use of enzyme-inducing drugs.^[27]^

### 4.3. Use of long-term TDM

TDM was used as part of the follow-up in all patients in this study. The pharmacokinetic variability was extensive within and between patients during a long period of time up to 8 years, and close monitoring with numerous changes in the treatment observed. Intra-patient variability could be more pronounced among the youngest patients with follow-up over several years. Many patients with TSC experience various treatment challenges, as they use different drugs in combination from a young age, have comorbidities including intellectual disabilities and may have difficulties in expressing possible adverse effects. In a clinical setting, tolerability may limit the use of everolimus due to immune system suppression, as it increases the risk of infections, especially in the upper respiratory tract.^[4,15,27]^ In the extension of EXIST-3, 249 patients were followed and included 149 responders of whom 70% were seizure free, and long-term effect lasted up to 1 year in two thirds.^[27]^ In another recent large study of 179 patients followed for 5 years, 118 reported at least 1 adverse effect, and this led to a dosage adjustment in one third of the patients.^[28]^ It is thus of importance to find the lowest possible effective dose, and TDM could be a useful tool to make such adjustments with a more proactive approach. Furthermore, a priori, dose and concentration relationships are unpredictable within the individual patients, but the optimal individual therapeutic concentration of the ASMs in each patient may be identified using TDM. This will allow the dose of everolimus to be adjusted to account for pharmacokinetic variation from, for example, enzyme-inducing ASMs.^[13,14,18,19]^ The present study demonstrated that the intra-patient variability was more extensive for everolimus than previously shown for other commonly used ASMs like valproate, clobazam, and levetiracetam, while inter-patient variability was in line with these drugs.^[14]^

### 4.4. Methodological considerations

In this study, we included a large proportion of patients with TSC in Norway and Denmark, and for most patients detailed information about the use and monitoring of ASMs was available. An advantage of this study is that we combine pharmacokinetic data with a clinical setting and elucidate the impact of detailed follow-up based on a recently published study of the same patient material.^[15]^ As retrospective routine measurements were used, no cross validation of the analyses between the laboratories was performed, but all 3 laboratories include external quality assurance control programs to ensure reliable and stable results. Body weight was not systematically noted for all patients at all time points, and thus C/D-ratios rather than C/D/kg were systematically used. Periodic or acute use of other drugs not noted could affect the metabolism of everolimus, such as CYP3A or P-glycoprotein transporter inhibitors, for example, the antibiotic drug clarithromycin could possibly affect C/D-ratios. In a realistic and retrospective setting, adherence could not be controlled for, but it is not assumed to be a major factor in this patient population.

## 5. Conclusions

This study demonstrates that there was extensive pharmacokinetic variability of everolimus in patients with TSC and in most cases, also epilepsy. The most common concomitant ASMs included lamotrigine, valproate, and levetiracetam, but 22% used enzyme inducers, which reduced the C/D-ratio of everolimus significantly, and the doses were not increased accordingly. The use of long-term TDM demonstrated unpredictable and extensive intra-patient and inter-patient pharmacokinetic variability in blood concentrations over time of about 40%. Long-term TDM may be used to follow and improve treatment outcome, taking comedication with enzyme inducers and overall pharmacokinetic variability into account.

## Acknowledgments

We would like to acknowledge Svein I. Johannessen, PhD, emeritus researcher, Margrete Larsen Burns, MD, PhD, Head of Section for Clinical Pharmacology, The National Center for Epilepsy, Oslo University Hospital, and Katrine Heger, MSc Pharm, PhD candidate at the Department of Pharmacy, Oslo Metropolitan University, for fruitful discussions of the present results, evaluations of statistical analyses and language improvements. We are also grateful to Nils Tore Vethe, PhD, Head of Section for Clinical Pharmacology, Oslo University Hospital for performing the analyses of everolimus in Norway as part of their routine service.

## Author contributions

**Data curation:** Kjersti Kirkeby, Christina Engel Hoei-Hansen, Lotte Holst, Mikkel G. Fredriksen, Cecilie Johanessen Landmark.

**Formal analysis:** Kjersti Kirkeby, Cecilie Johanessen Landmark.

**Investigation:** Kjersti Kirkeby, Ine Cockerell, Jakob Christensen, Christina Engel Hoei-Hansen, Lotte Holst, Mikkel G. Fredriksen, Caroline Lund, Cecilie Johanessen Landmark.

**Visualization:** Kjersti Kirkeby, Cecilie Johanessen Landmark.

**Writing – review & editing:** Kjersti Kirkeby, Ine Cockerell, Jakob Christensen, Christina Engel Hoei-Hansen, Lotte Holst, Mikkel G. Fredriksen, Caroline Lund, Cecilie Johanessen Landmark.

**Conceptualization:** Ine Cockerell, Jakob Christensen, Caroline Lund, Cecilie Johanessen Landmark.

**Methodology:** Jakob Christensen, Cecilie Johanessen Landmark.

**Validation:** Jakob Christensen, Caroline Lund, Cecilie Johanessen Landmark.

**Project administration:** Cecilie Johanessen Landmark.

**Supervision:** Cecilie Johanessen Landmark.

**Writing – original draft:** Cecilie Johanessen Landmark.
